# Do we understand the rationale behind driving restrictions in patients with an implantable cardioverter defibrillator?

**DOI:** 10.1007/s12471-017-1072-2

**Published:** 2018-01-12

**Authors:** S. W. E. Baalman, J. R. de Groot

**Affiliations:** Heart Center, Department of Cardiology, Academic Medical Center/University of Amsterdam, Amsterdam, The Netherlands

Patients with an implantable cardioverter defibrillator (ICD) have restrictions with regards to driving a motor vehicle. The Dutch Health Counsel initially recommended a 6-month driving restriction after ICD implantation for primary or secondary prevention of sudden cardiac death [1]. This was reduced to a 2-month restriction in 2004 [2], and recently even further in patients with a primary ICD indication to 2 weeks [3].

Driving restrictions in ICD patients are essentially based on two studies published in the late 1990 s. A survey by Curtis et al. found that 30 (10.5%) of 286 ICD shocks during driving resulted in accidents. However, the estimated injury and fatality rate for ICD patients was significantly lower than in the general population [4]. Jung et al. estimated the risk of harm (RH) to other road users or innocent bystanders posed by a driver implanted with an ICD, using the RH formula created by The Canadian Cardiovascular Society Consensus Conference [5, 6]. With the estimated annual risk of sudden cardiac death or incapacitation (SCI) as most important factor influencing this formula, the annual RH rate remained below the set cutoff value of 0.0005% in *private* ICD drivers but rose above the cutoff value in *commercial* drivers with an ICD. Subsequently, several risk of harm estimations have been published to establish evidence-based driving restrictions after ICD implantation or ICD shocks while driving [7]. This has led to a worldwide difference in driving restrictions in ICD patients [8].

As there is a lack of data regarding syncope symptoms in ICD patients while driving, the overall syncope rate in ICD patients has been used instead in most studies and RH calculations [8]. Also, recommendations are mostly based on older reports in a population of ICD patients with heart failure and coronary artery disease. As there are many other factors that may influence the risk of syncope or (in)appropriate shocks in ICD patients, how will the changing clinical profile of ICD patients influence these recommendations? For example, what will the risk of harm be of subcutaneous ICD patients, a relatively young population with less comorbidities? Or how about patients with a congenital heart disease or genetically determined arrhythmia syndrome, who experience a relatively high number of inappropriate shocks [9, 10]?

Limited ‘real world’ studies on the incidence of ICD shocks or syncope while driving, or car accidents in ICD patients have been published. Albert et al. examined the risk of ICD shocks while driving of patients enrolled in the TOVA study [11]. There was an increase in the incidence of shocks for VT or VF 30 min after, but not during driving. Akiyama et al. reported an annual accident rate in ICD patients that was lower than that in the general driving population in the US [12]. Eleven out of 171 (6%) of driving ICD patients were involved in car accidents as reported by Trappe et al. One of these 11 patients experienced an ICD shock due to VF, without any implications as the shock occurred after the accident [13]. In patients with a primary prevention indication, event rates tend to be even lower [14, 15]. In contrast, Bjerre et al. reported a 50% increase in traffic accidents involving ICD patients [16]. In that study the control group was only matched by age and gender and, although higher than the control group, the rate of car accidents in ICD patients was low without any observed deaths.

In this issue of the Netherlands Heart Journal, Jongejan et al. report a study regarding compliance of legal driving restrictions in Dutch ICD patients. For that, ICD patients completed a set of questionnaires, assessing driving-related characteristics at the time of implantation and after 4 months of follow-up [17]. Non-compliance of the driving restriction was reported by 28% of 313 patients. A multivariate analysis showed that the feeling of understanding the reason behind the driving restrictions was associated with better compliance.

Taken together, it seems that the risk of being involved in a car accident as ICD recipient is low, and that there are very limited data that having an ICD increases the risk of dying in a traffic accident. Jongejan et al. show us that patient understanding of the driving restrictions improves compliance, but our main question is: do we understand this legislation ourselves?
